# Brain ApoA-I, ApoJ and ApoE Immunodetection in Cerebral Amyloid Angiopathy

**DOI:** 10.3389/fneur.2019.00187

**Published:** 2019-03-13

**Authors:** Jessica Camacho, Teresa Moliné, Anna Bonaterra-Pastra, Santiago Ramón y Cajal, Elena Martínez-Sáez, Mar Hernández-Guillamon

**Affiliations:** ^1^Pathology Department, Vall d'Hebron University Hospital, Universitat Autònoma de Barcelona, Barcelona, Spain; ^2^Neurovascular Research Laboratory, Vall d'Hebron Research Institute, Universitat Autònoma de Barcelona, Barcelona, Spain

**Keywords:** ApoE, ApoA-I, ApoJ, clusterin, β-amyloid, cerebral amyloid angiopathy, intracerebral hemorrhage

## Abstract

Cerebral amyloid angiopathy (CAA) is a common cause of lobar intracerebral hemorrhage (ICH) in elderly individuals and it is the result of the cerebrovascular deposition of beta-amyloid (Aβ) protein. CAA is frequently found in patients with Alzheimer's disease (AD), although it has an independent contribution to the cognitive deterioration associated with age. Specific apolipoproteins (Apo) have been associated with Aβ fibrillization and clearance from the brain. In this regard, in the present study, we analyzed the brain levels of ApoE, ApoA-I, and ApoJ/clusterin in autopsy brains from 20 post-mortem cases with CAA type I, CAA type II, with parenchymal Aβ deposits or without Aβ deposits. Our objective was to find a possible differential pattern of apolipoproteins distribution in the brain depending on the CAA pathological presentation. The protein expression levels were adjusted by the APOE genotype of the patients included in the study. We found that ApoE and ApoJ were abundantly present in meningeal, cortical, and capillary vessels of the brains with vascular Aβ accumulation. ApoE and ApoJ also deposited extracellularly in the parenchyma, especially in cases presenting Aβ diffuse and neuritic parenchymal deposits. In contrast, ApoA-I staining was only relevant in capillary walls in CAA type I cases. On the other hand, ICH was the principal cause of death among CAA patients in our cohort. We found that CAA patients with ICH more commonly had APOEε2 compared with CAA patients without ICH. In addition, patients who suffered an ICH presented higher vascular ApoE levels in brain. However, higher ApoE presence in cortical arteries was the only independent predictor of suffering an ICH in our cohort after adjusting by age and APOE genotype. In conclusion, while ApoE and ApoJ appear to be involved in both vascular and parenchymal Aβ pathology, ApoA-I seems to be mainly associated with CAA, especially in CAA type I pathology. We consider that our study helps to molecularly characterize the distribution subtypes of Aβ deposition within the brain.

## Introduction

Cerebral beta-amyloidosis is defined as the anomalous deposition of β-amyloid (Aβ) protein in the brain. Aβ originates by the sequential processing of the amyloid precursor protein (APP), primarily generating peptides of 40 and 42 amino acids. The abnormal folding of amyloid material produces a β-pleated sheet secondary structure, which forms highly insoluble amyloid fibrils. In the most common form of Cerebral Amyloid Angiopathy (CAA), insoluble Aβ accumulates in the wall of the cortical and meningeal vessels, replacing the smooth muscle cells, and inducing vascular degeneration. However, Aβ can also accumulate in brain capillaries. In this regard, CAA can be neuropathologically classified as CAA type I, which is characterized by Aβ deposition in cerebral capillaries, or CAA type II, where cerebral capillaries are not involved (1). The most relevant clinical consequence of CAA is the risk of lobar symptomatic intracerebral hemorrhages (ICH) and microscopic cortical infarcts (2). CAA patients also present cognitive impairment, lower perceptual speed and episodic memory impairment, separately from the effect of Alzheimer's disease (AD) pathology (3). Indeed, AD is the most frequent neurodegenerative disease, morphologically characterized by the presence of hyperphosphorylated tau protein in the cytoplasm of neurons and extracellular Aβ deposits forming parenchymal plaques. Although CAA is observed in more than 90% of AD patients (4), only a minority of AD subjects demonstrate hemorrhages suggestive of advanced CAA (5). In fact, the combination of factors that determine whether Aβ accumulation occurs in parenchyma, developing into AD, or exclusively in blood brain vessels, triggering CAA-associated ICHs, still needs to be elucidated. It is plausible that the transport routes of Aβ will be crucial in this process. Evidence for that is the presence of vascular amyloid and the occurrence of cerebral microhemorrhages and vasogenic edema when parenchymal Aβ is mobilized by active or passive immunization in AD (6).

Specific apolipoproteins, beyond their natural role in cholesterol metabolism, have been proposed to be involved in Aβ transport and clearance from the brain (7, 8). Apolipoproteins represent the protein part of lipoproteins, which classically transport cholesterol from tissues to the liver. However, their presence and function in the brain are not well-described. The main apolipoproteins present in brain are apolipoprotein E (ApoE) and apolipoprotein J (ApoJ), both of them genetically associated with cerebral β-amyloidosis. On one hand, ApoE has a determining role in the progression of Aβ deposition, since having the APOEε4 allele is the major risk factor for AD (9), as well as for CAA (10). The role of ApoJ [also known as clusterin, (CLU)] in Aβ pathology is highlighted by genome-wide association studies (GWAS) that found a statistical association between a single nucleotide polymorphism within the CLU gene and the risk of suffering AD (11). In addition, more recently, the association between the CLU genotype and Aβ deposition, determined by PET imaging, has been described (12). Indeed, both ApoE and ApoJ have been shown to influence the amyloid fibrillogenesis process and clearance in experimental models (13–15). In this regard, apolipoprotein A-I (ApoA-I) has also emerged as a natural chaperone able to bind Aβ and prevent its aggregation and toxicity *in vitro* (16–18).

With this background, the aim of the present study was to immunohistochemically investigate the presence of ApoE, ApoJ, and ApoA-I in brains classified as CAA type I, CAA type II, brains with Aβ parenchymal deposits without vascular deposits and controls. Our hypothesis was that the expression and distribution of this family of apolipoproteins in the brain was modulated by the accumulation of Aβ as insoluble plaques, deposits in cortical/meningeal vessels or capillaries. Furthermore, we examined whether the immunopositivity of ApoE, ApoJ, and ApoA-I in brain vessels was associated with vascular Aβ deposition and symptomatic ICH appearance in patients affected by CAA.

## Materials and Methods

### Selection of Patients

**Figure 1:**
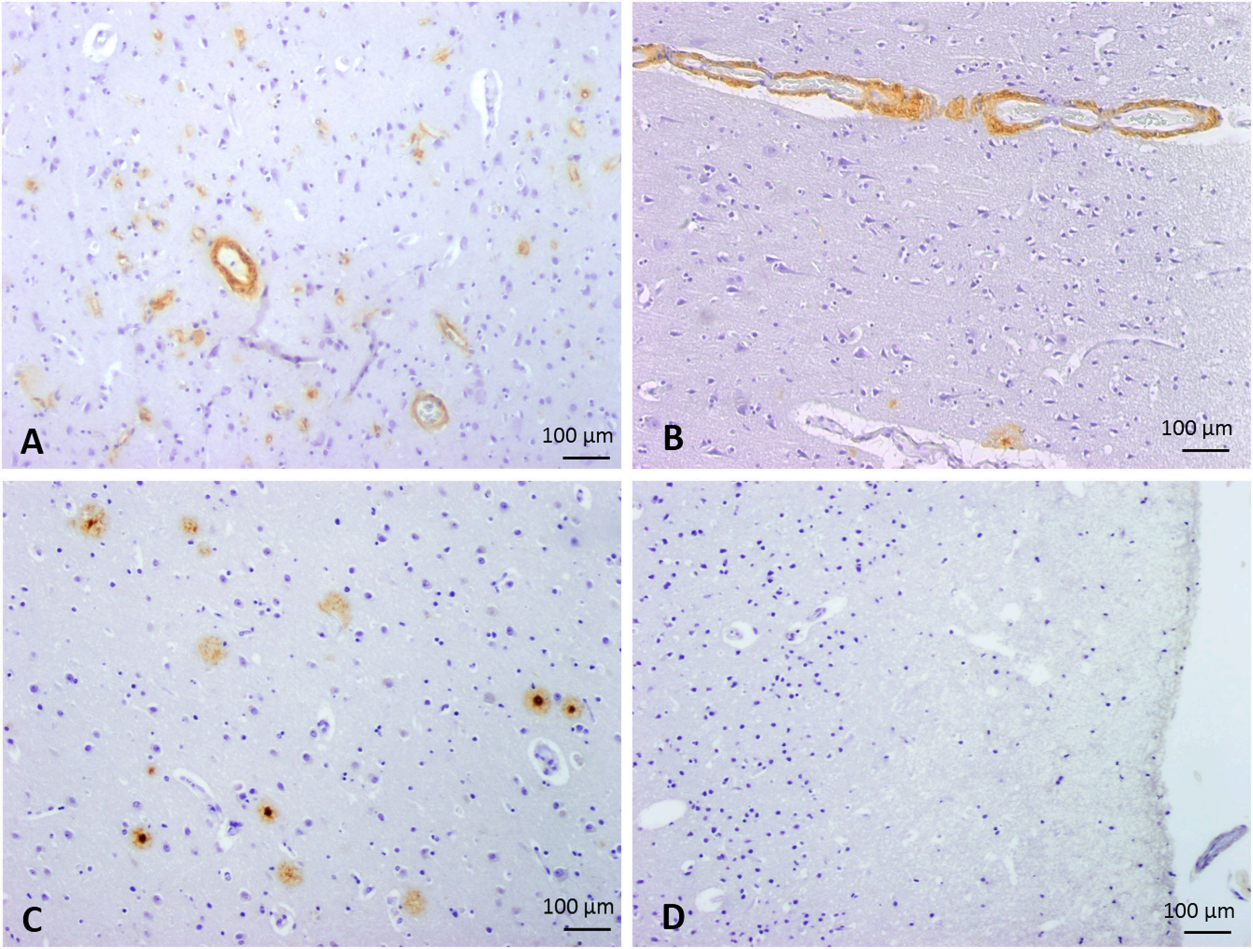
Classification of cases into 4 groups according to the parenchymal and vascular Aß deposition by immunostaining. Representative images of Aß immunohistochemistry. **(A)** Group 1: CAA type I, defined by the presence of capillary and arterial Aß deposits. **(B)** Group 2: CAA type II, defined by the presence of arterial Aß deposits without capillary involvement. **(C)** Group 3, characterized by the presence of parenchymal Aß deposits without vascular involvement. **(D)** Group 4, defined by the absence of Aß deposits (control cases). 100x magnification.

Twenty patients with a postmortem neuropathological study performed at the Pathology Department of Vall d'Hebron University Hospital were selected for the study. A written and informed consent was obtained from all patients (presenting Aß brain deposits), or their representatives, to participate in this research protocol before the tissue donation. For control cases, we received the exemption of the Ethic Committee to analyze them as anonymized samples in the context of this study. The project was approved by the Clinical Investigation Ethical Committee of the Vall d'Hebron University Hospital, Barcelona, Spain PR(IR)93/2013). Patients' characteristics and causes of death are presented in Table 1. All samples were obtained from 2 to 20 h after decease and post-mortem specimens were fixed in 10% buffered formalin for 3–4 weeks. Macroscopic examination was then performed, and 27 cortical and subcortical areas, including midbrain, and cerebellum, were selected for routine neuropathological diagnosis. Paraffin-embedded blocks were cut into 4 μm sections and stained with hematoxylin & eosin. Aß immunohistochemistry was performed in selected areas (as described at the end of this section). All cases were reviewed by two neuropathologists at a multiheaded microscope. Patients were divided into four groups according to Aß localization in the brain (Figure 1): CAA type I patients (group 1: 6/20 cases); CAA type II patients (group 2: 8/20 cases); patients with parenchymal Aß deposits without vascular involvement (group 3: 3/20 cases) and control cases with neither vascular nor parenchymal Aß deposits (group 4: 3/20 control cases).

**Table 1:** Cause of death and patients' characteristics.

**Case**	**Group**	**Age**	**Sex**	**APOE Genotype**	**Braak**	**Cerad**	**CAA type**	**Vonsattel**	**Cause of death**
1	1	85–90	F	E4/E3	V–VI	C	I	II	Intracerebral hemorrhage
2	1	75–80	F	E3/E3	IV	C	I	III	Intracerebral hemorrhage
3	1	75–80	F	E3/E3	I	A	I	II	Metastatic colon adenocarcinoma
4	1	70–75	M	E4/E3	II	B	I	II	Intracerebral hemorrhage
5	1	80–85	M	E4/E2	–	–	I	II	Intracerebral hemorrhage
6	1	75–80	M	E4/E3	III	C	I	II	Heart failure
7	2	80–85	M	E2/E3	IV	B	II	II	Intracerebral hemorrhage
8	2	85–90	F	E2/E3	V–VI	C	II	IV	Intracerebral hemorrhage
9	2	65–70	M	E3/E3	II	C	II	II	Pericarditis
10	2	90–95	F	E3/E3	III	C	II	I	Mesenteric thrombosis
11	2	75–80	M	E4/E3	I	B	II	II	Metastatic prostate adenocarcinoma
12	2	80–85	F	E2/E3	III	A	II	II	Intracerebral hemorrhage
13	2	80–85	F	E3/E3	–	–	II	I	Secondary amyloidosis
14	2	70–75	F	E3/E3	–	–	II	I	Metastatic breast carcinoma
15	3	75–80	F	E3/E3	I	A	–	–	Acute meningitis
16	3	55–60	F	E3/E3	II	C	–	–	Ischemic stroke
17	3	75–80	F	E3/E3	I	B	–	–	Acute pneumonia
18	4	50–55	M	E4/E3	–	–	–	–	Pulmonary hypertension
19	4	60–65	F	E3/E3	–	–	–	–	Large B cell lymphoma
20	4	70–75	M	E4/E3	–	–	–	–	Multiple Myeloma

*F, female; M, male*.

CAA lesions were graded following the criteria described by Vonsattel et al. (19) and modified by Greenberg and Vonsattel (20). Grade 0 CAA was given when no amyloid could be found in the vessel wall. Grade 1 CAA lesions showed amyloid deposits in otherwise normal vessels. The thickening of the vessel wall with a stiff appearance defined a grade 2 CAA lesion, where the media was replaced by amyloid. Grade 3 CAA showed cracking of the vessel wall, creating a “vessel-within-vessel” appearance affecting at least 50% of the circumference of the vessel. In grade 4 CAA lesions, scarring of the wall was observed, with fibrinoid necrosis and traces of intermingled amyloid deposits. Classification into CAA type I and CAA type II was made according to the criteria described by Thal et al. (presence and absence of capillary Aß deposits in types I and II, respectively) (1). Alzheimer disease pathology was evaluated and staged according to Braak (21) and CERAD's criteria (22) through immunohistochemistry using the mouse anti-human Amyloid-ß Clone 6F/3D 1:500 (Dako, Glostrup, Denmark) and the mouse anti-human Tau protein Clone AT8 1:20 (Thermo Scientific, Rockford, USA).

### Immunohistochemistry

To describe a representative distribution of Aβ deposits, up to 3 blocks from cortical areas, including perihippocampal cortex in five cases, were selected in order to evaluate an equivalent number of meningeal and cortical vessels (20–30 per case). Brainstem and cerebellar areas were not used in this study. Consecutive sections of the selected blocks were performed for the detection of ApoE, ApoJ, and ApoA-I. In those samples from patients who suffered an ICH, the immunopositivity of the different apolipoproteins were evaluated outside the lesion.

Paraffin was removed, endogenous peroxidases were blocked with methanol-H_2_O_2_, and non-specific binding sites were blocked with 10% normal goat serum for 1 h. Sections were incubated with mouse anti-human ApoE 1:200 (Clone E6D7, Calbiochem—NE1004, Madrid, Spain), mouse anti-human ApoJ 1:1,000 (Clone 5 BD Pharmingen −552886, Madrid, Spain) and rabbit anti-human ApoA-I 1:200 (Clone EP1368Y, Abcam—ab52945, Cambridge, UK) for 40 min at RT. Secondary antibody (goat anti-mouse horseradish peroxidase, 1:500) was applied for 8 min. Immunoreactive sites were developed with DAB solution and serial sections were stained with hematoxylin & eosin (H&E) to assess morphology. Meningeal and cortical vessels, neurons, subpial astrocytes, and parenchymal deposits in cortical and perihippocampal areas were semi-quantitatively evaluated. Blind to the group distribution, two neuropathologists assessed the three antibodies under a multiheaded microscope until agreement was reached.

### ApoE, ApoJ, and ApoA-I Immunopositivity Scores

Supplemental Table 1 summarizes the scores used for the vascular and parenchymal immunodetection. The presence of ApoE, ApoJ, and ApoA-I immunostaining in cortical and meningeal arteries was graded according to the percentage of arteries showing immunopositivity: score 0 was given when no positive arteries could be found; score 1 indicated <30% of positive arteries; score 2 corresponded to 30–75% of positive arteries; and score 3 indicated immunopositivity in more than 75% of the evaluated arteries. ApoE, ApoJ, and ApoA-I presence in cortical capillaries was evaluated in 10 high-power field (HPF) and scored as follows: score 0 when no signal was detected in cortical capillaries; score 1 when the signal was focal (in fewer than 50% of the capillaries); score 2 when the signal was focal and linear (in fewer than 50% of the capillaries); score 3 when the signal was diffuse (in more than 50% of capillaries); and score 4 when the signal was strong and thick in the capillaries. ApoE, ApoJ, and ApoA-I parenchymal deposits were evaluated in 10 HPF, after detecting the hotspot for each block, and graded according to the number of parenchymal deposits, divided into neuritic-like plaques (with a dense core surrounded by a clear halo and, sometimes, a neuritic corona) and diffuse-like plaques (without any central core): score 0 indicated absence of parenchymal deposits; score 1 indicated sparse deposits (<5/HPF); score 2 indicated a moderate number of deposits (5–10/ HPF); and score 3 indicated frequent deposits (>10/ HPF). Immunodetection of ApoE, ApoJ, and ApoA-I in the cytoplasm of subpial astrocytes and pyramidal neurons was semi-quantitatively rated as: absence, mild, moderate, or intense expression.

### APOE Genotype

Genomic DNA was extracted from formalin-fixed, paraffin-embedded tissue using EZ1 DNA Tissue kit (48) and Bio Robot EZ1 (Qiagen, Hilden, Germany). APOE genotypes (rs429358 and rs7412) were determined by allelic discrimination using the SNP genotyping mixes C-3084793 and C-904973 (Applied Biosystems, Foster City, CA, USA) and the TaqMan_Genotyping Master Mix (Applied Biosystems) in a Rotor Gene 6000 Real-Time PCR analyzer (Corbett Life Sciences, Valencia, CA, USA).

### Statistical Analysis

Statistical analysis was conducted using SPSS package 17.0. Clinical or pathological associations with age (continuous variable following a normal distribution after applying the Kolmogorov–Smirnov test) were tested using the Student's *t*-test or One-way ANOVA test, as appropriate. The rest of the variables analyzed were considered categorical variables and the significance of frequency differences were calculated using the Chi Square (χ^2^) test or the exact test of Fisher for 2 × 2 contingency tables. Logistic regression analysis were performed to identify independent associations with the apolipoproteins expression. A *p* < 0.05 was considered statistically significant.

## Results

ApoE, ApoJ, and ApoA-I cerebral levels and localization were analyzed in brains from the four different groups of patients categorized according to the parenchymal and vascular Aß deposition determined by immunostaining (Figure 1). Principal demographic characteristics, AD, and CAA pathological scores and APOE genotype of all patients included in the study can be found in Table 1. CAA cases (encompassing groups 1 and 2) were significantly older than non-CAA cases (encompassing groups 3 and 4) (81 ± 7 vs. 66 ± 12, *p* = 0.026). As expected, ICH was the cause of death in CAA patients more often than in non-CAA cases [50% (7/14) vs. 0% (0/6), *p* = 0.036]. Among CAA patients, no differences were found in age (*p* = 0.983), sex (*p* = 0.774), or cause of death between CAA type I (group 2) and type II (group 1) cases (*p* = 0.298). However, APOEε4 genotype was more represented in CAA type I patients than in those lacking capillary CAA (type II) [67% (4/6) vs. 13% (1/8), *p* = 0.036]. CAA type II cases had a slightly higher ε2 allele frequency than CAA type I, although differences were not significant [38% (3/8) vs. 17% (1/6), *p* = 0.393]. APOE genotype was not statistically associated with sex, age or other pathological scores. However, APOE genotype was associated with having suffered an ICH (*p* = 0.017). Interestingly, CAA patients with ICH had a significantly higher APOEε2 genotype frequency compared with CAA patients without ICH [57% (4/7) vs. 0% (0/7), *p* = 0.018].

**Figure 2:**
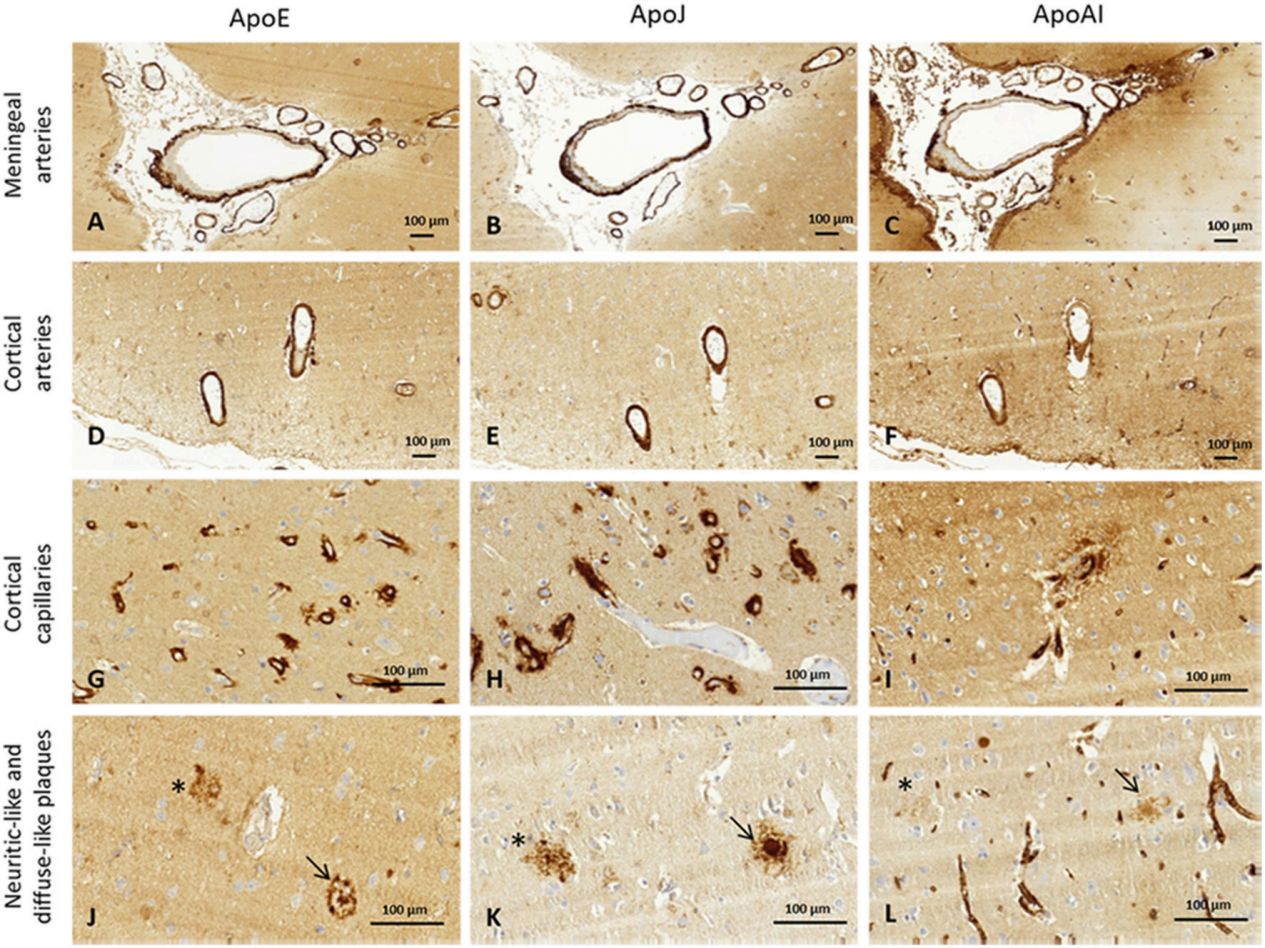
Representative images of brain ApoE, ApoJ, and ApoA-I immunopositivity. Serial sections were stained with anti-ApoE **(A,D,G,J)**, anti-ApoJ **(B,E,H,K)**, and anti-ApoA-1 antibodies **(C,F,I,L)**. Detection of these proteins were found in meningeal vessels **(A–C)**, cortical vessels **(D–F)**, cortical capillaries **(G–I)**, and diffuse-like (asterisks) and neuritic-like plaques (arrows) **(J–L)**. Meningeal and cortical vessels: 100x magnification. Neuritic plaques and capillaries: 400x magnification.

The presence of ApoE, ApoJ, and ApoA-I in cortical and meningeal arteries, capillaries, neuritic-like, and diffuse parenchymal deposits was evaluated by immunohistochemistry. Examples of immunopositivity for each case are shown in Figure 2. Parenchymal ApoE, ApoJ, and ApoA-I presence was also analyzed within brain cells (Supplemental Figure 1).

### ApoE, ApoJ, and ApoA-I Presence in Cerebral Arteries

**Figure 3:**
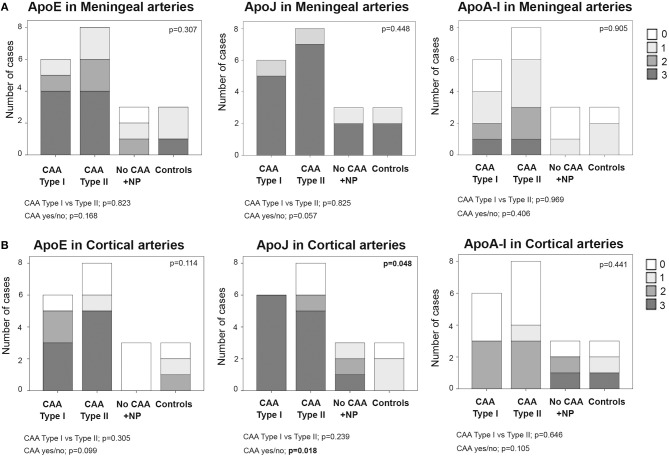
Distributions of ApoE, ApoJ and ApoA-I in meningeal **(A)** and cortical **(B)** arteries. 0: No staining; 1: staining in <30% of the vessels; 2: staining in 30–75% of the vessels; 3: staining in >75% of the vessels. Statistical differences were calculated using the Chi Square (χ2) test.

In meningeal vessels, ApoE positivity was not significantly different among groups (*p* = 0.307): score 3 was given to 67% (4/6) of CAA type I, 50% (4/8) of CAA type II, and 33% (1/3) of control cases. ApoJ levels in meningeal vessels were higher than ApoE but was equally represented in all groups (*p* = 0.448). The highest ApoJ level score (score 3) was given to 83% (5/6) of CAA type I cases, 87% (7/8) of CAA type II cases, 67% (2/3) of brains with parenchymal Aß deposits without Aß vascular involvement and 67% (2/3) of control cases. ApoA-I showed a lower presence in meningeal vessels than ApoE or ApoJ and did not differ among groups (*p* = 0.905); most of the samples received scores of 1 or 0. Expression of ApoE, ApoJ, and ApoA-I in the meningeal arteries was not significantly different between CAA type I and CAA type II cases or between CAA and non-CAA cases (Figure 3A, Supplemental Table 2). APOE genotype was not associated with the expression of ApoE, ApoJ, and ApoA-I in meningeal arteries.

Regarding the presence of these apolipoproteins in cortical arteries, ApoE showed a score of 3 in 50% (3/6) of CAA type I and 63% (5/8) of CAA type II cases. Interestingly, cortical vessels from brains with parenchymal Aß deposits without vascular Aß involvement were not positive for ApoE (all cases scored 0), but the differences among the 4 groups did not reach statistical significance (*p* = 0.114). ApoJ vascular cortical positivity was higher than ApoE, showing a score of 3 in all (100%, 6/6) CAA type I cases, in 63% (2/3) of CAA type II cases and in 33% (1/3) of brains with parenchymal Aß deposits without vascular involvement. ApoJ expression in the cortical vessels of controls was lower than in those cases with cerebral Aß accumulation (*p* = 0.048). Presence of ApoA-I was also less frequent in cortical vessels and was similarly distributed among groups (*p* = 0.441). Expression of ApoE, ApoJ, and ApoA-I in cortical arteries was not significantly different between CAA type I and CAA type II cases. However, CAA brains presented a higher percentage of ApoE-positive cortical vessels than non-CAA cases (*p* = 0.099), and this was especially relevant for ApoJ (*p* = 0.018) (Figure 3B, Supplemental Table 2). In addition, the presence of ApoE in cortical arteries was nearly significantly related to the APOEε2 genotype (*p* = 0.058).

### ApoE, ApoJ, and ApoA-I Presence in Brain Cortical Capillaries

**Figure 4:**
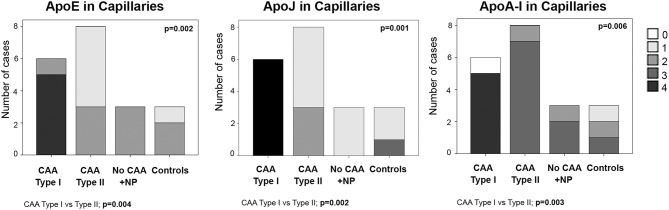
Distributions of ApoE, ApoJ, and ApoA-I in cortical capillaries. 0: No staining; 1: focal and granular wall staining (<50% of the capillaries); 2: focal and linear wall staining (<50% of the capillaries); 3: diffuse wall staining (>50% of the capillaries); 4: strong staining. Statistical differences were calculated using the Chi Square (χ2) test.

ApoE staining in the capillaries scored 4 in 83% (5/6) of CAA type I cases (group 1), in which the presence was higher than in the other groups (*p* = 0.002). In fact, ApoE expression in brain capillaries was significantly more abundant in CAA type I cases than in CAA type II cases (*p* = 0.004). ApoJ positivity in the capillaries followed the same pattern as ApoE: 100% (6/6) of CAA type I cases presented the highest score in ApoJ levels (score 4), whereas the mean score was lower in the other groups (*p* = 0.001). ApoJ presence was also stronger in microvessels affected by Aß accumulation, so levels were higher in brain capillaries from CAA type I cases than from CAA type II cases (*p* = 0.002). Interestingly, ApoA-I positivity in the capillaries was more pronounced globally and more homogenous between groups, although microvessels from CAA type I samples also presented the highest score in 83% (5/6) of the cases, which was more often than the other groups (*p* = 0.006). Indeed, ApoA-I levels in brain capillaries were also significantly higher in CAA type I cases than in CAA type II cases (*p* = 0.003) (Figure 4, Supplemental Table 2). In addition, potential associations between ApoE, ApoJ, and ApoA-I presence in brain capillaries and APOE genotype were determined. In this regard, only the expression of ApoE protein in capillaries was related to APOEε4 genotype (*p* = 0.035), although the association did not achieve statistical significance after the adjustment by age and CAA subtype (odds ratio (OR) 2.32; 95% confidence interval (CI) [0.97–5.87], *p* = 0.073).

### ApoE, ApoJ, and ApoA-I Parenchymal Accumulation

**Figure 5:**
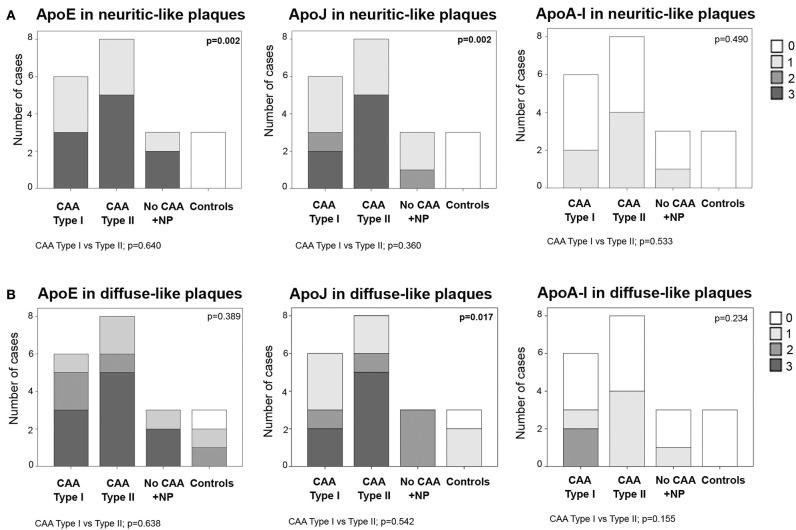
Distribution of ApoE, ApoJ and ApoA-I in neuritic plaques **(A)** or diffuse parenchymal deposits **(B)**. 0: No staining; 1: 1-5/HPF; 2: 5-10/HPF; 3: >10/HPF. Statistical differences were calculated using the Chi Square (χ2) test.

Frequent ApoE neuritic-like plaques (score 3) were found in 50% (3/6) of CAA type I cases, 63% (5/8) of CAA type II cases and 67% (2/3) of cases with parenchymal Aß deposits without vascular involvement. No ApoE neuritic-like plaques were found in control cases (differences among the 4 groups: *p* = 0.002). Similarly, 33% (2/6) of CAA type I and 63% (5/8) of CAA type II cases showed frequent ApoJ neuritic-like plaques (score 3), whereas there were lower scores in cases with parenchymal Aß deposits without vascular involvement and no ApoJ neuritic-like plaques in control cases (*p* = 0.002). ApoA-I deposits were almost absent in all of the analyzed samples, and the distribution was not different among groups (*p* = 0.490). No differences were found in the number of ApoE, ApoJ, or ApoA-I neuritic-like plaques between CAA type I and type II cases (Figure 5A, Supplemental Table 2). APOE genotype was not associated with the presence of ApoE, ApoJ, and ApoA-I forming neuritic or diffuse-like parenchymal deposits

Frequent ApoE diffuse-like plaques (score 3) were found in 50% (3/6) of CAA type I cases, in 63% (5/8) of CAA type II cases and 67% (2/3) of cases with parenchymal Aß deposits without vascular Aß involvement. Brain samples from controls presented fewer ApoE diffuse-like plaques, although differences among the 4 groups were not statistically significant (*p* = 0.389). The presence of ApoJ forming diffuse-like plaques was frequent (score 3) in 33% (2/6) of CAA type I and 63% (5/8) of CAA type II cases, in comparison with a lower detection in the rest of the groups (*p* = 0.017). Although ApoE and ApoJ diffuse-like plaques were found in 67% (2/3) of controls, groups 3 and 4 presented lower scores compared with CAA groups 1 and 2 in both cases. ApoA-I diffuse-like plaques were globally less frequent than ApoE and ApoJ, without significant differences among groups (*p* = 0.234). Presence of ApoE, ApoJ, or ApoA-I in diffuse-like plaques was also not different between CAA type I and type II cases (Figure 5B, Supplemental Table 2). APOE genotype was not associated with the presence of ApoE, ApoJ, and ApoA-I forming neuritic or diffuse-like parenchymal deposits in our cohort.

ApoE, ApoJ, and ApoA-I were expressed in the cytoplasm of pyramidal neurons and subpial astrocytes from different cortical and perihippocampal areas analyzed. No differences in the number of positive cells nor in the intensity of the expression were detected for ApoE, ApoJ, or ApoA-I among the 4 groups analyzed or between CAA type I and CAA type II cases. ApoE, ApoJ, and ApoA-I immunopositivity in pyramidal neurons and subpial astrocytes are presented in Supplemental Figure 1.

### Vascular ApoE, ApoJ, and ApoA-I Levels Associated With ICH

**Figure 6:**
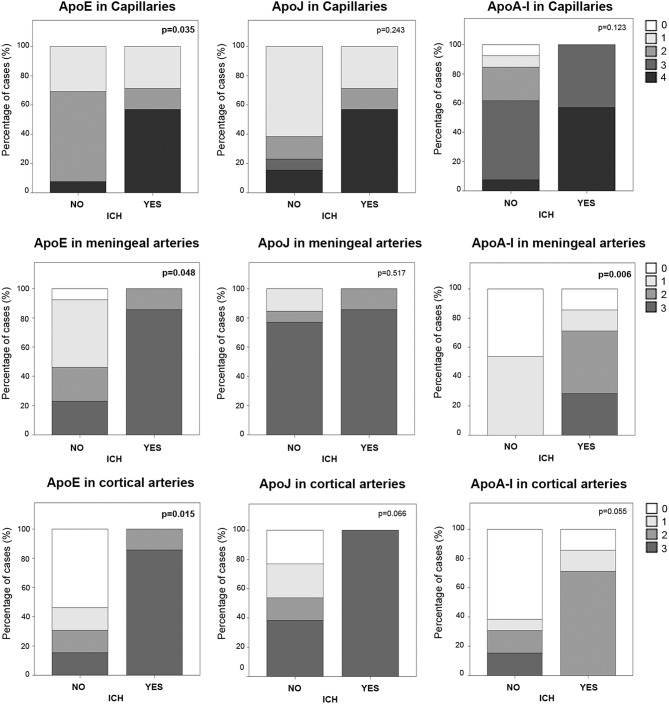
Distribution of ApoE, ApoJ, and ApoA-I in capillaries and meningeal and cortical arteries in brains from subjects who had an intracerebral hemorrhage (ICH) or not. Graphs represent the percentage of samples in each group (presenting ICH or not). The presence in brain capillaries was classified as 0: no staining; 1: focal and granular wall staining (<50% of the capillaries); 2: focal and linear wall staining (<50% of the capillaries); 3: diffuse wall staining (>50% of the capillaries); 4: strong staining. The presence in brain arteries was classified as 0: no staining; 1: staining in <30% of the vessels; 2: staining in 30–75% of the vessels; 3: staining in >75% of the vessels. Statistical differences were calculated using the Chi Square (χ2) test.

In the cohort of analyzed postmortem brains, lobar ICH was the principal cause of death among CAA patients (*N* = 7, Table 1). Sex was not associated with ICH presence, but subjects who suffered an ICH were older than the others (83 ± 6 vs. 73 ± 11, *p* = 0.040). We evaluated the possible association between the levels of ApoE, ApoJ, and ApoA-I in brain vessels and the occurrence of a parenchymal bleeding, although apolipoprotein presence was evaluated far from the acute hematoma in ICH cases. In this regard, ApoE was significantly higher in capillaries and in meningeal and cortical arteries in those patients who suffered an ICH (*p* = 0.035, *p* = 0.048, and *p* = 0.015, respectively) (Figure 6), although ApoE immunodetection was not associated with age. In contrast, ApoE levels in diffuse or neuritic-like plaques were not different in patients who suffered an ICH. ApoA-I levels in meningeal arteries were higher in ICH patients than in the other subjects (*p* = 0.006). Finally, ApoJ parenchymal presence was not statistically associated with ICH in this cohort (Figure 6). Nevertheless, after adjusting for age and APOE genotype, higher ApoE presence in cortical arteries was the only independent predictor of suffering an ICH (OR, 9.40, 95% CI [1.05–84.41], *p* = 0.046).

## Discussion

It is still not completely clear why Aβ accumulates in the brain in certain neuropathological conditions, such as AD and CAA. Although certain inherited forms of CAA presenting cerebral hemorrhage are associated with autosomal dominant mutations in APP and other genes [including the cystatin C (CST3), integral transmembrane protein 2B (ITM2B), transthyretin and gelsolin genes] in most cases, sporadic CAA does not clearly associate with any genetic risk factor other than the association described for APOEε4 (10, 23). One of the most accepted hypotheses that tries to explain the sporadic cerebrovascular Aβ deposition refers to “protein elimination failure” due to a deficit in protein aggregate clearance mechanisms (24). The cerebrovasculature is the main system responsible for the clearance of cerebral Aβ, which is evacuated through perivascular drainage routes and excluded through the blood-brain barrier (BBB). The fibrillar and compact Aβ deposition in the brain vessel walls has been related to abnormalities in the vasculature, which can subsequently compromise the integrity of the BBB. Indeed, the most relevant pathological consequence of CAA is the development of ICHs affecting cortical and subcortical areas. Furthermore, recurrence is a common complication in CAA patients, which results in elevated mortality and disability rates (25). Nevertheless, the mechanisms of vessel rupture triggering ICH due to Aβ deposition have not been clarified either. In this regard, our aim was to study the presence of factors that codeposit with Aβ in brains showing different Aβ localization patterns. In particular, we analyzed specific apolipoproteins, ApoE, ApoJ, and ApoA-I, which may influence the insoluble deposit aggregation and clearance (13–16) and therefore can be relevant in the distribution of Aβ in the brain.

Brain ApoE is produced by glia (astrocytes and microglia), pericytes, smooth muscle cells, and, to a much lesser extent, by neurons under certain stress conditions and, potentially, in brain endothelial cells. ApoE modulates multiple mechanistic pathways that affect cognition, including cholesterol/lipid homeostasis, synaptic function, glucose metabolism, neurogenesis, mitochondrial function, tau phosphorylation, neuronal atrophy, neuroinflammation, and the metabolism and aggregation of Aβ (26). Previous immunohistochemical studies in AD brains revealed the presence of ApoE in parenchymal deposits and in the wall of brain vessels, where the ApoE level correlated with Aβ deposits (8, 27, 28). Our results confirm some of these previous findings, as we detected higher parenchymal ApoE levels in brains where a previous presence of Aβ, forming neuritic plaques, was confirmed. However, no significant differences were found in the ApoE level in meningeal arteries affected or not by CAA, although a tendency toward a higher ApoE presence was observed in cortical vessels from CAA brains than those from non-CAA brains.

Several evidences have demonstrated that APOE genotype modifies the distribution of cerebrovascular Aβ in AD individuals (23, 29). In this regard, we studied whether the APOE genotype could also influence the expression of ApoE, ApoJ, and ApoA-I in CAA cases. Although APOEε4 is a well-known risk factor for CAA (10, 23), we could not show this association in our study, probably because of the small sample size of the control group (cases without Aβ pathology). However, among CAA cases, we found that APOEε4 genotype was more represented in CAA type I patients, whereas CAA type II patients seemed to present higher APOEε2 frequency, in accordance with previous studies (1, 30). No significant association could be found between the immunodetection of ApoE, ApoJ, or ApoA-I and the APOE genotype, except for a statistical trend pointing toward a link between APOE ε2 genotype and higher presence of ApoE in cortical arteries. In fact, in CAA-associated ICH patients, our group previously showed higher plasma ApoE protein levels in APOEε2 carriers (31). On the other hand, the presence of either APOEε4 or APOEε2 have been described to increase the risk of lobar ICH (32). Previous pathological studies demonstrated that APOEε2 may promote the CAA-related vessel breakdown (33), whereas APOEε4 was associated with the amount of vascular amyloid (10). Interestingly, in our cohort, CAA patients with ICH more commonly were APOEε2 carriers compared with CAA patients without ICH, validating the results described previously (34). In addition to this, the presence of ApoE in cortical vessels was significantly higher in patients who suffered an ICH, after adjusting for APOE genotype and age. This data suggests the possible role of this apolipoprotein in the destabilization and disruption of brain vessels, as proposed in different experimental models (35, 36).

ApoJ is a multifunctional heterodimeric protein that acts as a natural chaperone. ApoJ binds to Aβ in CSF and plasma (37), and increased plasma ApoJ has been associated with a higher prevalence and severity of AD (38). In AD brains, ApoJ codeposits with Aβ in cerebrovascular and parenchymal lesions (27, 39, 40). Once again, the results from our study are in accordance with the published data, since we clearly confirmed higher ApoJ levels in brain vessels from CAA cases and in parenchymal neuritic-like and diffuse-like deposits in cases presenting Aβ plaques. However, it is worth mentioning that ApoJ and ApoE diffuse-like deposits were also found in some control cases, where no deposits of Aβ were detected in parenchyma. The use of more sensitive immunodetection methods (for example, using Aβ antibodies that recognize different epitopes) would be required to confirm these results. The increased levels of ApoJ has been postulated as a protective response against the aberrant accumulation of Aβ in the brain. However, there are conflicting results in the literature regarding the protective role of ApoJ. Knocking out *CLU* in a transgenic mouse model of AD resulted in an exacerbation of vascular Aβ deposition, whereas parenchymal Aβ deposition was remarkably reduced (41). In another study, intraventricular administration of an ApoJ-mimetic peptide promoted a reduction in parenchymal and vascular Aβ accumulation and improved cognitive function in an experimental AD model (42). Thus, the impact of the alteration of the ApoJ level in the brain still needs to be clarified. In our study, in contrast to ApoE, no significant association was found between ApoJ cerebrovascular levels and the occurrence of ICH.

Finally, ApoA-I is a major constituent of high-density lipoprotein (HDL) in plasma and plays a crucial role in reverse cholesterol transport. It is produced mainly in the liver and intestine, but ApoA-I is considered to be able to cross the BBB (43) because it has been found in CSF and throughout the brain (44), although the mechanisms behind this trafficking are not well-described. In fact, in AD brains, ApoA-I has been associated with senile plaques (8, 27), although it is not clear whether this plaque-associated ApoA-I comes from the periphery or whether it is expressed by specific brain cells, such as endothelial capillary cells (45). In the present study, ApoA-I was also found in occasional brain parenchymal deposits. However, ApoA-I positivity was less diffuse than ApoE and ApoJ in meningeal and cortical arteries, and there was no association between ApoA-I levels and Aβ arterial deposition.

Another important aim of our study was to determine whether these codeposition factors were present when cases were classified according to Aβ capillary involvement. Indeed, the clinical relevance of the CAA neuropathological classification is under investigation. For instance, one study found that CAA type I seems to be associated with the severity of CAA, dementia and severe AD-type neuropathology (46), while another study reported that the associations of CAA with cognitive outcomes were not driven by the presence of capillary involvement (47). In our study, CAA capillary involvement was not associated with the CAA or AD pathological severity. CAA subtype was not related to ICH occurrence either. On the other hand, the biological pattern of both CAA entities has not been studied extensively (48). In this context, the expression of these apolipoproteins in cerebral vessels affected by Aβ deposition was previously described (27, 28), although no specific reports have described a possible differential distribution pattern depending on the CAA pathological classification. Therefore, we determined the levels and localizations of ApoE, ApoJ, and ApoA-I in brains classified as CAA type I or type II. We found that the presence of the three apolipoproteins in capillaries from CAA cases was more evident than in those from non-CAA cases. Furthermore, the endothelial expression levels of ApoE and ApoJ, as well as those of ApoA-I, were higher in the CAA type I capillaries than in the CAA type II capillaries. In fact, endothelial expression with strong and thick wall staining and, sometimes, perivascular deposits was found in CAA type I capillaries, while only endothelial expression without significant wall staining was found in CAA type II capillaries. Interestingly, our results highlight the relevant specific association between ApoA-I presence and capillary Aβ, although whether this ApoA-I has a peripheral origin and is attracted by the microvascular Aβ or this is a specific expression mechanism in endothelial cells needs to be elucidated. Furthermore, higher levels of ApoE and ApoA-I were also detected in capillaries from patients who suffered an ICH, independently of the CAA subtype, suggesting the possible involvement of the accumulation of these two apolipoproteins in the alteration of some properties associated to the BBB integrity.

In summary, ApoE and ApoJ appear to be involved in both vascular and parenchymal Aβ pathology, while ApoA-I seems to be mainly associated with CAA, especially in CAA type I. In addition, ApoE presence in cortical arteries appears as an independent factor for the occurrence of ICH in CAA. Our study presents important limitations, such as the small sample size of the subgroups (principally evident for the non-CAA groups) or the analysis of different cortical areas, which may have influenced the apolipoprotein immunodetection rating. Further studies are needed to evaluate the impact of the co-deposition of these apolipoproteins in Aβ-related pathologies and in the occurrence of CAA-associated ICH.

## Author Contributions

MH-G and EM-S designed and coordinated the study. JC participated in the design of the study. SR advised on the theoretical aspects of the study. TM performed the immunohistochemistry analysis. JC and EM-S selected brain samples and revised all the immunohistochemistry analysis until agreement was reached. AB-P performed the APOE genotype analysis from paraffin-embedded sections. JC, EM-S, and MH-G wrote the manuscript. All authors read and approved the final manuscript.

### Conflict of Interest Statement

The authors declare that the research was conducted in the absence of any commercial or financial relationships that could be construed as a potential conflict of interest.
