# Supplementary material for: Brain ApoA-I, ApoJ and ApoE Immunodetection in Cerebral Amyloid Angiopathy
Source: Front Neurol. 2019 Mar 13;10:187. doi: 10.3389/fneur.2019.00187 (PMC6424885; doi:10.3389/fneur.2019.00187)
Supplement: Supplementary file 1 [file Table_1.DOCX]

**Supplemental Material**

**Supplemental Table 1.** ApoE, ApoJ and ApoA-I immunopositivity scores

|  | **Cortical capillaries** | **Meningeal and cortical vessels** | **Parenchymal deposits** |
| --- | --- | --- | --- |
| **0** | No staining | No staining | No staining |
| **1** | Focal and granular wall staining (<50% of the capillaries) | Staining of <30% of the vessels evaluated | Sparse deposits  (<5/ high-power field) |
| **2** | Focal and linear wall staining (<50% of the capillaries) | Staining 30-75% of the vessels evaluated | Moderate number of deposits  (5-10/ high-power field) |
| **3** | Diffuse wall staining (> 50% of capillaries) | Staining >75% of the vessels evaluated | Frequent deposits  (>10/ high-power field) |
| **4** | Strong and thick staining in the capillaries |  |  |

**Supplemental Figure 1.** Representative images of ApoE, ApoJ and ApoA-I immunopositivity in subpial astrocytes and pyramidal neurons. Detection of ApoE (A, D), ApoJ (B,E) and ApoA-I (C,F) were found in subpial astrocytes cytoplasm (A-C) and pyramidal neurons cytoplasm (D-F). 400x magnification.

**
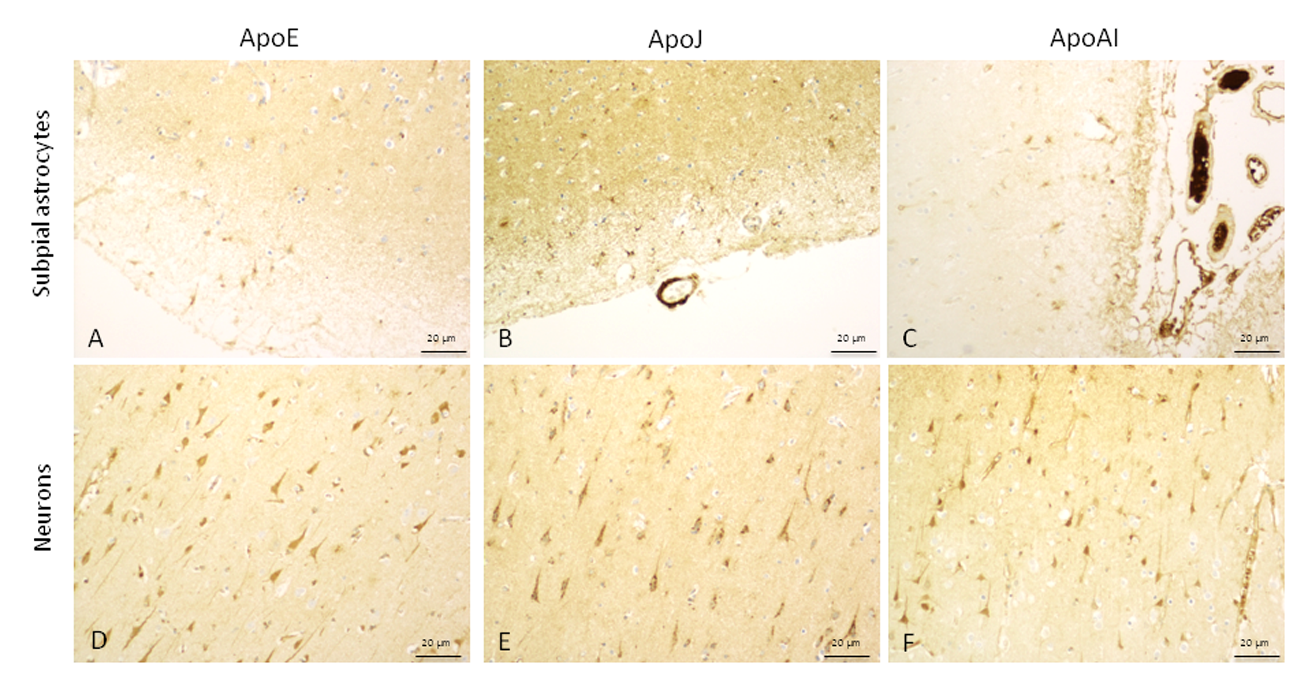
**

|  | | **ApoE** | | | | **ApoJ** | | | | **ApoA-I** | | | | |
| --- | --- | --- | --- | --- | --- | --- | --- | --- | --- | --- | --- | --- | --- | --- |
|  | | **CAA**  **Type I** | **CAA**  **Type II** | **No CAA/ Aβ dep.** | **controls** | **CAA**  **Type I** | **CAA**  **Type II** | **No CAA/**  **Aβ dep.** | **controls** | **CAA**  **Type I** | **CAA**  **Type II** | **No CAA/ Aβ dep.** | | **controls** |
| **Capillary staining** | **0** | 0/6 (0%) | 0/8 (0%) | 0/3 (0%) | 0/3 (0%) | 0/6 (0%) | 0/8 (0%) | 0/3 (0%) | 0/3 (0%) | 1/6 (17%) | 0/8 (0%) | 0/3 (0%) | | 0/3 (0%) |
|  | **1** | 0/6 (0%) | 5/8 (63%) | 0/3 (0%) | 1/3 (33%) | 0/6 (0%) | 5/8 (63%) | 3/3 (100%) | 2/3 (67%) | 0/6 (0%) | 0/8 (0%) | 0/3 (0%) | | 1/3 (33%) |
|  | **2** | 1/6 (17%) | 3/8 (37%) | 3/3 (100%) | 2/3 (67%) | 0/6 (0%) | 3/8 (37%) | 0/3 (0%) | 0/3 (0%) | 0/6 (0%) | 1/8 (13%) | 1/3 (33%) | | 1/3 (33%) |
|  | **3** | 0/6 (0%) | 0/8 (0%) | 0/3 (0%) | 0/3 (0%) | 0/6 (0%) | 0/8 (0%) | 0/3 (0%) | 1/3 (33%) | 0/6 (0%) | 7/8 (87%) | 2/3 (67%) | | 1/3 (33%) |
|  | **4** | 5/6 (83%) | 0/8 (0%) | 0/3 (0%) | 0/3 (0%) | 6/6 (100%) | 0/8 (0%) | 0/3 (0%) | 0/3 (0%) | 5/6 (83%) | 0/8 (0%) | 0/3 (0%) | | 0/3 (0%) |
| p value  between groups | | **0.002** | | | | **0.001** | | | | **0.006** | | | | |
| p value CAA I vs CAAII | | **0.004** | | | | **0.002** | | | | **0.003** | | | | |
| p value CAA yes/no | | *0.066* | | | | **0.033** | | | | 0.145 | | | | |
| **Meningeal arteries** | **0** | 0/6 (0%) | 0/8 (0%) | 1/3 (33%) | 0/3 (0%) | 0/6 (0%) | 0/8(0%) | 0/3 (0%) | 0/3 (0%) | 2/6 (33%) | 2/8 (25%) | 2/3 (67%) | | 1/3 (33%) |
|  | **1** | 1/6 (17%) | 2/8 (25%) | 1/3 (33%) | 2/3 (67%) | 0/6 (0%) | 0/8(0%) | 1/3 (33%) | 1/3 (33%) | 2/6 (33%) | 3/8 (37%) | 1/3 (33%) | | 2/3 (67%) |
|  | **2** | 1/6 (17%) | 2/8 (25%) | 1/3 (33%) | 0/3 (0%) | 1/6 (17%) | 1/8 (12%) | 0/3 (0%) | 0/3 (0%) | 1/6 (17%) | 2/8 (25%) | 0/3 (0%) | | 0/3 (0%) |
|  | **3** | 4/6 (67%) | 4/8 (50%) | 0/3 (0%) | 1/3 (33%) | 5/6 (83%) | 7/8 (87%) | 2/3 (67%) | 2/3 (67%) | 1/6 (17%) | 1/8 (13%) | 0/3 (0%) | | 0/3 (0%) |
| p value  between groups | | 0.307 | | | | 0.448 | | | | 0.905 | | | | |
| p value CAA I vs CAAII | | 0.823 | | | | 0.825 | | | | 0.969 | | | | |
| p value CAA yes/no | | 0.168 | | | | *0.057* | | | | 0.406 | | | | |
| **Cortical**  **arteries** | **0** | 1/6 (17%) | 2/8 (25%) | 3/3 (100%) | 1/3 (33%) | 0/6 (0%) | 2/8 (25%) | 0/3 (0%) | 1/3 (33%) | 3/6 (50%) | 4/8 (50%) | 1/3 (33%) | | 1/3 (33%) |
|  | **1** | 0/6 (0%) | 1/8 (13%) | 0/3 (0%) | 1/3 (33%) | 0/6 (0%) | 0/8 (0%) | 1/3 (33%) | 2/3 (67%) | 0/6 (0%) | 1/8 (13%) | 0/3 (0%) | | 1/3 (33%) |
|  | **2** | 2/6 (33%) | 0/8 (0%) | 0/3 (0%) | 1/3 (33%) | 0/6 (0%) | 1/8 (13%) | 1/3 (33%) | 0/3 (0%) | 3/6 (50%) | 3/8 (37%) | 1/3 (33%) | | 0/3 (0%) |
|  | **3** | 3/6 (50%) | 5/8 (63%) | 0/3 (0%) | 0/3 (0%) | 6/6 (100%) | 5/8 (63%) | 1/3 (33%) | 0/3 (0%) | 0/6 (0%) | 0/8(0%) | 1/3 (33%) | | 1/3 (33%) |
| p value  between groups | | 0.114 | | | | **0.048** | | | | 0.441 | | | | |
| p value CAA I vs CAAII | | 0.305 | | | | 0.239 | | | | 0.646 | | | | |
| p value CAA yes/no | | *0.099* | | | | **0.018** | | | | 0.105 | | | | |
| **Neuritic**  **plaques** | **0** | 0/6 (0%) | 0/8 (0%) | 0/3 (0%) | 3/3 (100%) | 0/6 (0%) | 0/8 (0%) | 0/3 (0%) | 3/3 (100%) | 4/6 (67%) | 4/8 (50%) | 2/3 (67%) | | 3/3 (100%) |
|  | **1** | 3/6 (50%) | 3/8 (37%) | 1/3 (33%) | 0/3 (0%) | 3/6 (50%) | 3/8 (38%) | 2/3 (67%) | 0/3 (0%) | 2/6 (33%) | 4/8 (50%) | 1/3 (33%) | | 0/3 (0%) |
|  | **2** | 0/6 (0%) | 0/8 (0%) | 0/3 (0%) | 0/3 (0%) | 1/6 (17%) | 0/8 (0%) | 1/3 (33%) | 0/3 (0%) | 0/6 (0%) | 0/8 (0%) | 0/3 (0%) | | 0/3 (0%) |
|  | **3** | 3/6 (50%) | 5/8 (63%) | 2/3 (67%) | 0/3 (0%) | 2/6 (33%) | 5/8 (63%) | 0/3 (0%) | 0/3 (0%) | 0/6 (0%) | 0/8 (0%) | 0/3 (0%) | | 0/3 (0%) |
| p value  between groups | | **0.002** | | | | **0.002** | | | | 0.490 | | | | |
| p value CAA I vs CAAII | | 0.640 | | | | 0.360 | | | | 0.533 | | | | |
| **Diffuse**  **plaques** | **0** | 0/6 (0%) | 0/8 (0%) | 0/3 (0%) | 1/3 (33%) | 0/6 (0%) | 0/8(0%) | 0/3 (0%) | 1/3 (33%) | 3/6 (50%) | 4/8 (50%) | | 2/3 (67%) | 3/3 (100%) |
|  | **1** | 1/6 (17%) | 2/8 (25%) | 1/3 (33%) | 1/3 (33%) | 3/6 (50%) | 2/8 (25%) | 0/3 (0%) | 2/3 (67%) | 1/6 (17%) | 4/8 (50%) | | 1/3 (33%) | 0/3 (0%) |
|  | **2** | 2/6 (33%) | 1/8 (13%) | 0/3 (0%) | 1/3 (33%) | 1/6 (17%) | 1/8 (13%) | 3/3 (100%) | 0/3 (0%) | 2/6 (33%) | 0/8(0%) | | 0/3 (0%) | 0/3 (0%) |
|  | **3** | 3/6 (50%) | 5/8 (63%) | 2/3 (67%) | 0/3 (0%) | 2/6 (33%) | 5/8 (63%) | 0/3 (0%) | 0/3 (0%) | 0/6 (0%) | 0/8(0%) | | 0/3 (0%) | 0/3 (0%) |
| p value  between groups | | 0.389 | | | | **0.017** | | | | 0.234 | | | | |
| p value CAA I vs CAAII | | 0.638 | | | | 0.542 | | | | 0.155 | | | | |

**Supplemental Table 2.** ApoE, ApoJ and ApoA-I immunodetection in the different subgroups. Statistical differences were calculated using the Chi Square (χ2) test.
